# Lamins and nesprin-1 mediate inside-out mechanical coupling in muscle cell precursors through FHOD1

**DOI:** 10.1038/s41598-017-01324-z

**Published:** 2017-04-28

**Authors:** Christine Schwartz, Martina Fischer, Kamel Mamchaoui, Anne Bigot, Thevy Lok, Claude Verdier, Alain Duperray, Richard Michel, Ian Holt, Thomas Voit, Suzanna Quijano-Roy, Gisèle Bonne, Catherine Coirault

**Affiliations:** 1 Sorbonne Universités, UPMC Univ Paris 06, INSERM UMRS974, CNRS FRE3617, Centre for Research in Myology, Paris, France; 2 Univ. Grenoble Alpes, LIPHY, F-38000 Grenoble, France; 3 CNRS, LIPHY, F-38000 Grenoble, France; 4 INSERM, Institut Albert Bonniot, U1209, F-38000 Grenoble, France; 5 Université Grenoble Alpes, IAB, F-38000 Grenoble, France; 6 Wolfson Centre for Inherited Neuromuscular Disease, RJAH Orthopaedic Hospital, Oswestry, SY10 7AG UK; 7 APHP, GHU Paris Ile-de-France Ouest, Garches, France; 8 NIHR Great Ormond Street Biomedical Research Centre, Institute of Child Health, University College London, 30 Guilford Street, London, WC1N 1EH UK

## Abstract

LINC complexes are crucial for the response of muscle cell precursors to the rigidity of their environment, but the mechanisms explaining this behaviour are not known. Here we show that pathogenic mutations in *LMNA* or *SYNE-1* responsible for severe muscle dystrophies reduced the ability of human muscle cell precursors to adapt to substrates of different stiffness. Plated on muscle-like stiffness matrix, mutant cells exhibited contractile stress fibre accumulation, increased focal adhesions, and higher traction force than controls. Inhibition of Rho-associated kinase (ROCK) prevented cytoskeletal defects, while inhibiting myosin light chain kinase or phosphorylation of focal adhesion kinase was ineffective. Depletion or inactivation of a ROCK-dependent regulator of actin remodelling, the formin FHOD1, largely rescued morphology in mutant cells. The functional integrity of lamin and nesprin-1 is thus required to modulate the FHOD1 activity and the inside-out mechanical coupling that tunes the cell internal stiffness to match that of its soft, physiological-like environment.

## Introduction

Cells fine tune their cytoskeleton tension to match the stiffness of the microenvironment, a process which may have a profound effect on the forces transmit to the nucleus. The cytoskeleton provides a network that physically couples the cell periphery to the nuclear envelope (NE). Cytoskeletal tension generated by actomyosin interactions along actin filaments is transduced across the NE via linker of nucleoskeleton and cytoskeleton (LINC) complexes^1–3^. Members of the LINC complexes include the giant protein nesprins and the SUN proteins that bind via their nucleoplasmic domains to A-type lamins^4^. LINC complexes span the NE and physically link the nucleoskeleton and the cytoskeleton. Together LINC complexes and the A-type lamins play crucial roles in different function including nucleo-cytoskeletal coupling, nuclear positioning^5^ and mechanotransduction^6^.

The integrity of nuclear-cytoskeletal linkages is particularly crucial for muscle function^7–10^. Mutations in genes encoding nesprins-1 and -2^8, 11–14^, SUN proteins^15, 16^ or A-type lamins^17^ cause muscular dystrophies. To date, all mutations in A-type lamins^18, 19^ or nesprins^9, 20, 21^ that cause striated muscle disease compromise the nesprin/SUN/lamin interactions, resulting in dysfunctional nucleo-cytoskeletal linkages^9, 10, 16, 18, 20, 22^.

Although detailed mechanisms remain to be determined, there is growing evidence that dysfunctional LINC complexes can in turn impair the dynamics and organization of the actin cytoskeleton^7, 23–25^. Functional loss in A-type lamins alters cytoskeletal actin structures around the nucleus in cells cultured on a rigid substrate^25–27^, presumably through an impaired activation of the mechanosensitive transcriptional cofactor myocardin-related transcription factor A/serum responsive factor and its target genes^28^. A-type lamin mutations also compromise the ability of cells to adapt their actin cytoskeleton to a soft 3D environment and to withstand mechanical stretching of the ECM, owing to the deregulation of Yes-Associated Protein (YAP) signalling pathways^29^. Collectively, these results implicate LINC complexes in modulating the dynamics and organization of the actin cytoskeleton and thus the mechanosensing response. However, previous studies do not identify the specific actin regulatory proteins involved in this defective actin remodelling.

Among a rich variety of regulators, the diaphanous related formins (DRF), encoded by the *DIAPH* genes, constitute a family of Rho-GTPase-regulated proteins that regulate actin and microtubule cytoskeleton remodelling^30^. Formins affect actin polymerisation and depolymerisation in a force-sensitive manner^31, 32^. Recent data indicate that formin FHOD1 is associated with dorsal actin cables and co-localizes with Transmembrane Actin associated Nuclear (TAN) lines via binding to the giant nesprin-2 isoform^33^, thus suggesting that dysfunction of nuclear-cytoskeletal linkages may modulate the perinuclear actin network through FHOD1 activity.

To determine how mutations known to alter the functional integrity of LINC complexes affect the ability of muscle cell precursors to match their cytoskeleton tension to the stiffness of the microenvironment, we have used human myoblasts with *LMNA* ^29, 34^ or *SYNE-1* mutations^35, 36^ (hereafter named *LMNA* ^ΔK32^ and Nespr-1^ΔKASH^ myoblasts, respectively) responsible for severe muscular dystrophies. Here, we demonstrate that the functional integrity of A-type lamin and nesprin-1 is required for myoblasts to adapt to the rigidity of their physiological matrix. Indeed, in a soft environment close to physiological muscle stiffness, we found that myoblasts with *LMNA* and *SYNE-1* mutations exhibited increased actin cytoskeletal assembly, increased focal adhesion formation, reduced nucleus thickness and increased traction force. In addition, we provide evidence that the underlying mechanism for this phenotype involved the activation of the formin FHOD1, presumably through an increased ROCK activity. Our results strongly suggest that nuclear-cytoskeletal linkages regulate a feedback loop that tunes internal stiffness of the cells to match that of their soft microenvironment, through inside to outside pathways involving the actin cytoskeleton and the formin FHOD1.

## Results

### Impaired adaptation to substrate stiffness in Nespr-1^ΔKASH^ and LMNA^ΔK32^ myoblasts

**Figure 1:**
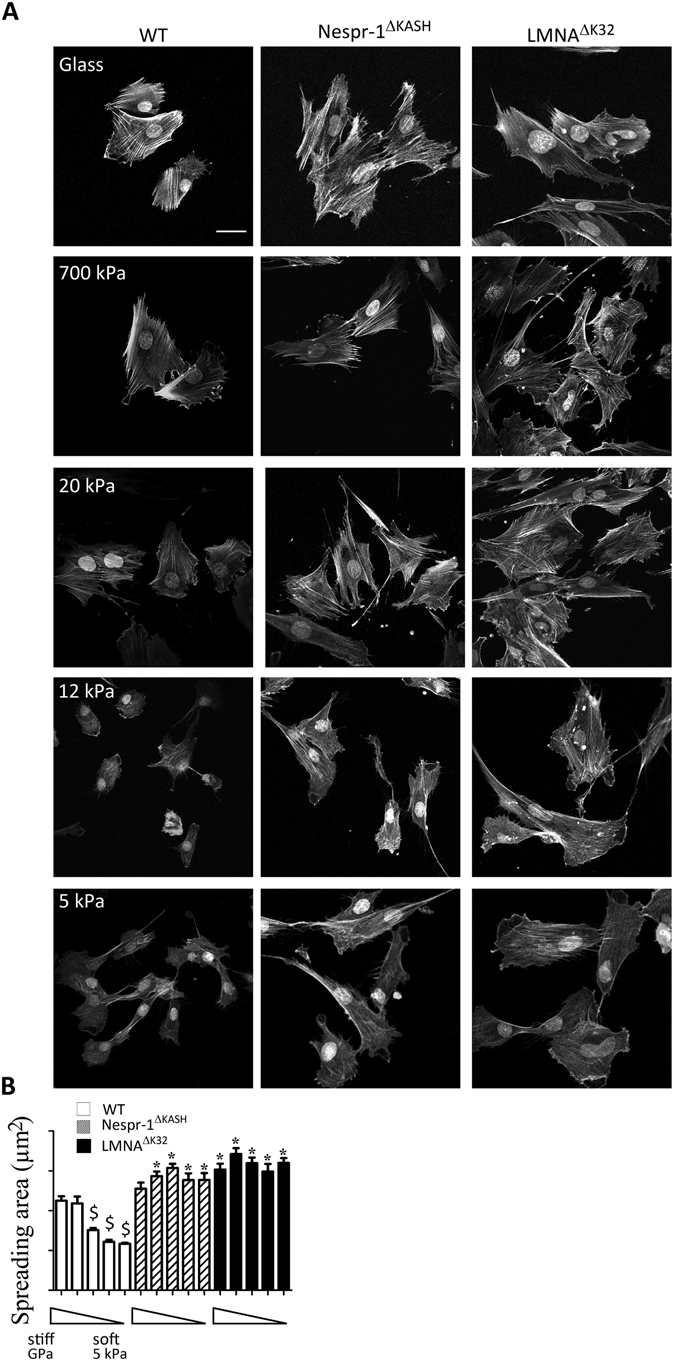
Cell responses to different substrate stiffness. (**A**) Phalloidin staining of the F-actin of fixed WT, Nespr-1^ΔKASH^ and LMNA^ΔK32^ myoblasts on fibronectin-coated glass and gel substrates of 700 kPa, 20 kPa, 12 kPa and 5 kPa. Nuclei are stained with DAPI. Scale bar: 40 µm. (**B**) Projected cell area as a function of substrate stiffness. Analysis was performed on glass and gel substrates of 700 kPa, 20 kPa, 12 kPa, and 5 kPa (each n > 50 cells). Values are means ± SEM; ^$^p < 0.001 vs corresponding cell line value on glass; *p < 0.001 vs WT value at similar substrate rigidity.

Using fibronectin-coated glass (~GPa) and hydrogels of known rigidity ranging from 5 kPa to 700 kPa, we first investigated the ability of WT Nespr-1^ΔKASH^ and LMNA^ΔK32^ myoblasts to adapt to the stiffness of their surrounding substrates. As expected, the spreading of WT cells, reflected by the total cell area, significantly decreased with substratum rigidity from 700 kPa to 5 kPa (Fig. 1A,B). In contrast, Nespr-1^ΔKASH^ and LMNA^ΔK32^ myoblasts did not modulate their spreading with substratum rigidity (Fig. 1A,B). These results show that Nespr-1^ΔKASH^ and LMNA^ΔK32^ myoblasts fail to adapt to their mechanical environment in a range of stiffness spanning that of muscle tissue^37^.

### Increased contractility of Nespr-1^ΔKASH^ and LMNA^ΔK32^ myoblasts on matrix stiffness close to that of muscle

#### Contractile actin stress fibre accumulation in mutant cells

**Figure 2:**
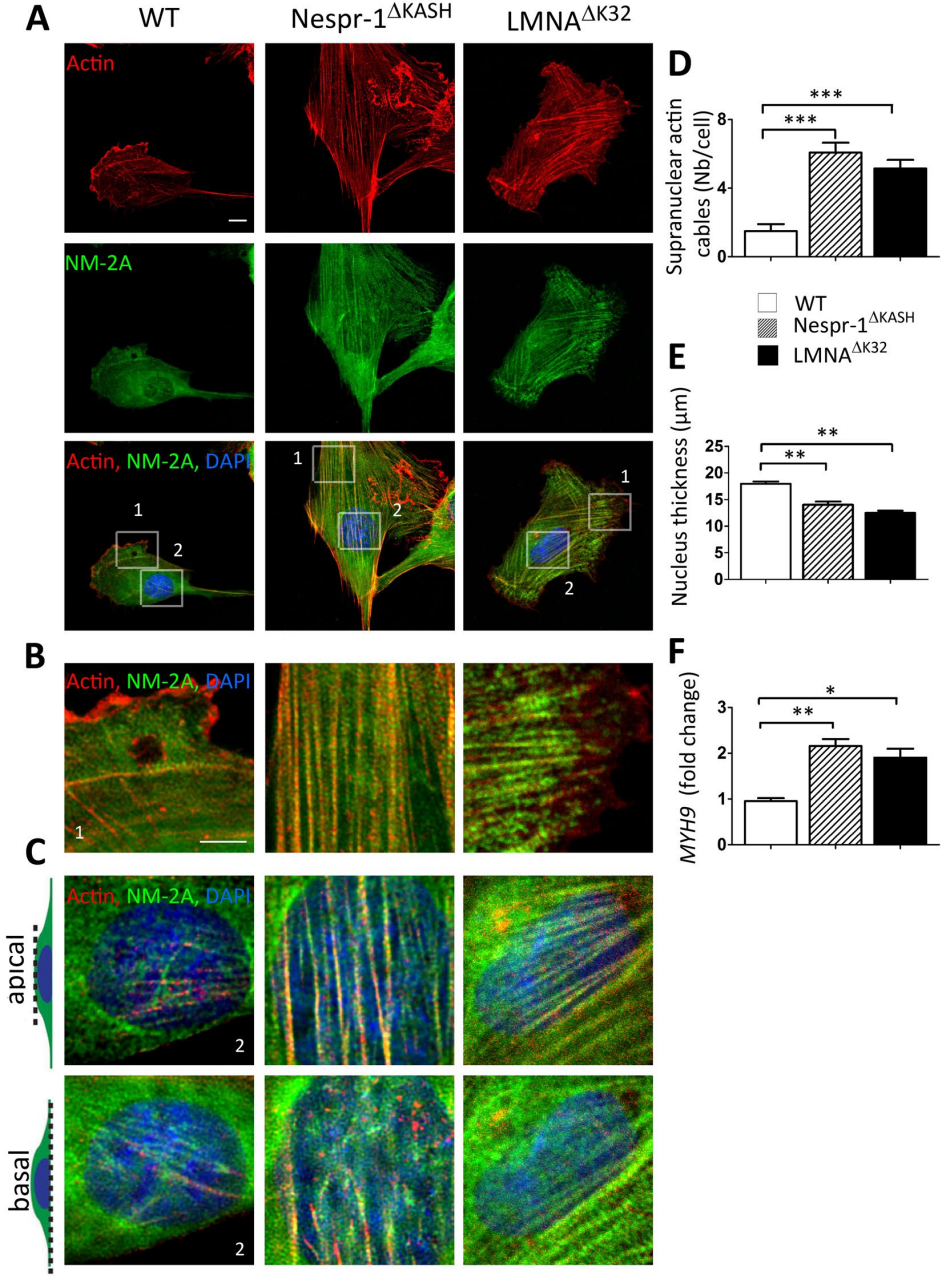
Actin cytoskeleton on soft matrix (12 kPa). (**A**) Confocal images of WT, Nespr-1^ΔKASH^ and LMNA^ΔK32^ myoblasts on soft matrix (12 kPa) close to physiological muscle stiffness and stained for F-actin (phalloidin, red) and non-muscle myosin 2 A (NM-2A, green). Nuclei are stained with DAPI (blue). Scale bar: 10 µm. (**B**,**C**) Zoom-in of actin cytoskeleton at the cell periphery (**B**) and in the perinuclear regions (**C**). In C, confocal images are taken at the apical and basal surface of the cell. Scale bar: 5 µm. (**D**) Supranuclear actin cable number in WT, Nespr-1^ΔKASH^ and LMNA^ΔK32^ myoblasts. Values are means ± SEM; ***p < 0.001 compared with WT. (**E**) Nuclear thickness in WT, Nespr-1^ΔKASH^ and LMNA^ΔK32^ myoblasts. Values are means ± SEM; **p < 0.01 compared with WT. (**F**) mRNA expression of *MYH9* gene expression coding for NM-2A in WT, Nespr-1^ΔKASH^ and LMNA^ΔK32^ myoblasts. Values are means ± SEM; **p < 0.01, *p < 0.05 compared with WT.

We next sought to investigate the contractile actin cytoskeleton organization in Nespr-1^ΔKASH^ and LMNA^ΔK32^ myoblasts cultured on matrix stiffness close to that of muscle, i.e., 12 kPa^37^. We found clear modifications in the organization of the actomyosin stress fibres in mutant cells compared with WT (Fig. 2A–D). As expected, WT myoblasts on 12 kPa displayed only few convex shaped contractile fibres at the cell periphery that resembled transverse arcs^38^. In contrast, both Nespr-1^ΔKASH^ and LMNA^ΔK32^ myoblasts had numerous thick actomyosin bundles, present both at the cell periphery and in the nuclear and perinuclear regions (Fig. 2B–D). These contractile stress fibres could extend throughout most of the cell length, thus resembling ventral stress fibres^38^. Importantly, they were present both at the basal and apical surfaces of the mutant cells (Fig. 2C), with a reduction in nuclear height in both Nespr-1^ΔKASH^ and LMNA^ΔK32^ compared with WT nuclei (Fig. 2E). The nuclear volume did not differ between WT and mutant nuclei (Suppl Fig. 1A), suggesting that different mechanisms controlled nuclear volume and nuclear thickness. In addition, the mRNA expression of *MYH9*, the gene encoding non-muscle myosin 2 A (NM-2A) was significantly up-regulated in Nespr-1^ΔKASH^ and LMNA^ΔK32^ compared with WT (Fig. 2F). These results show that Nespr-1^ΔKASH^ and LMNA^ΔK32^ myoblasts accumulate contractile stress fibres when plated in conditions close to their physiological stiffness.

#### Increased maturation of focal adhesions in mutant cells

**Figure 3:**
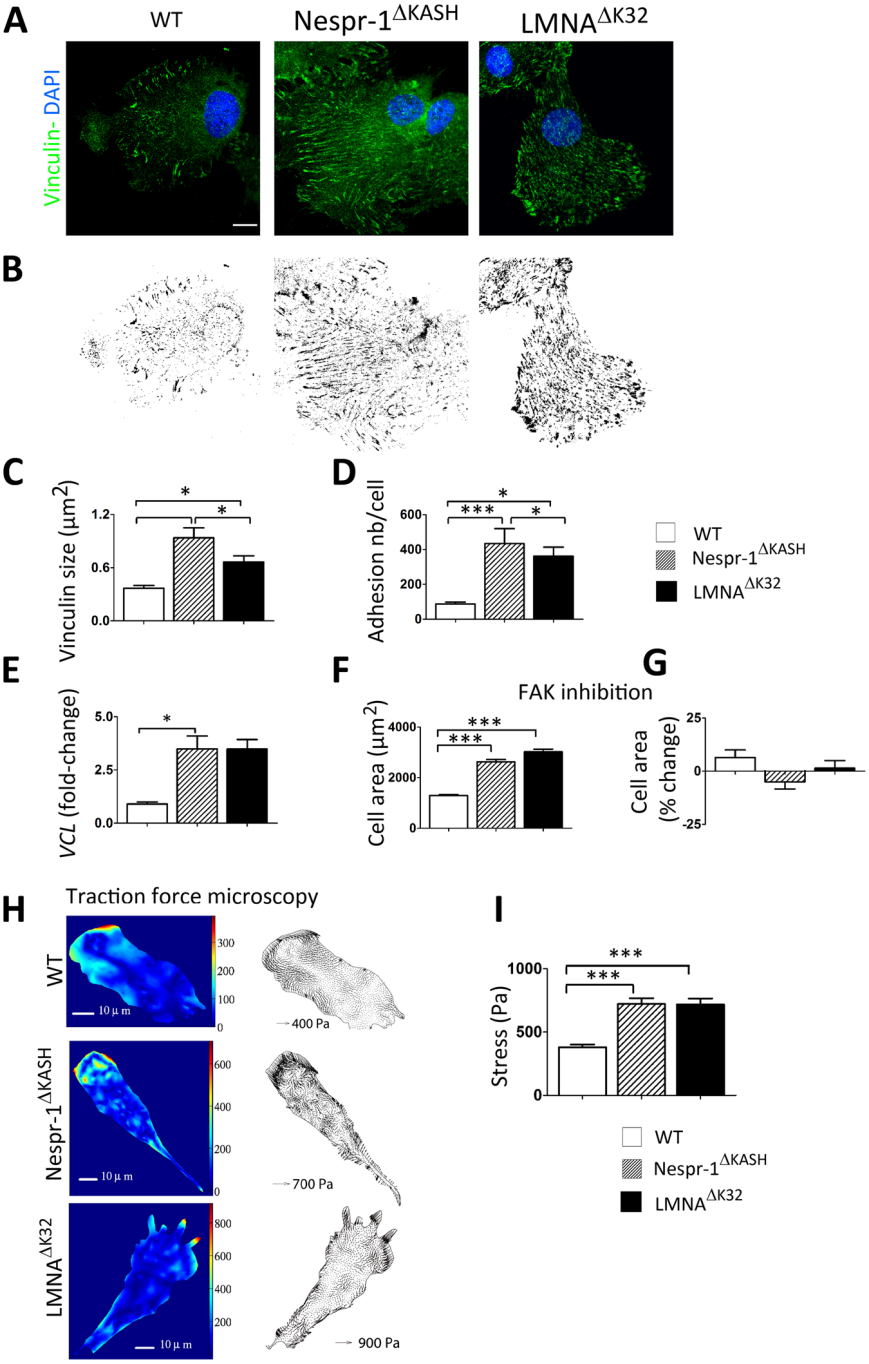
Cell matrix adhesions on soft matrix (12 kPa). (**A**) Confocal images of WT, Nespr-1^ΔKASH^ and LMNA^ΔK32^ myoblasts on soft matrix (12 kPa) and stained with antibody against vinculin (green). Nuclei are stained with DAPI (blue). Scale bar: 10 µm. (**B**) Binary images of vinculin staining obtained from A. (**C**,**D**) Quantification of vinculin size (**C**) and number of focal adhesions per cell (**D**) in WT, Nespr-1^ΔKASH^ and LMNA^ΔK32^ myoblasts obtained from binary images. Values are means ± SEM in at least 12 myoblasts per line; *p < 0.05, **p < 0.01, ***p < 0.001 compared with WT. (**E**) Histogram of *VCL* mRNA expression in WT, Nespr-1^ΔKASH^ and LMNA^ΔK32^ myoblasts. Values are means ± SEM; *p < 0.05 compared with WT and expressed in arbitrary units (au). Values are means ± SEM, n = 5 in each cell line from 2 separate experiments. (**F**,**G**) Effects of FAK phosphorylation inhibition on cell spreading in WT, Nespr-1^ΔKASH^ and LMNA^ΔK32^ myoblasts. Values are expressed as µm^2^ (**F**) and as percent of baseline values for each cell line (**G**). At least 50 cells of each type were measured, ***p < 0.001 compared with WT. (**H**,**I**) Traction force microscopy in WT, Nespr-1^ΔKASH^ and LMNA^ΔK32^ myoblasts. (**H**) Typical images of traction isostresses (Pa) and traction stress vectors (Pa) in WT, Nespr-1^ΔKASH^ and LMNA^ΔK32^ myoblasts. (**I**) Mean values ± SEM obtained from n ≥ 9 in each cell line; ***p < 0.001 compared with WT.

To test whether changes in stress fibre formation were accompanied by changes in focal adhesions, we next examined vinculin, a scaffolding protein that contributes to mechanosensitivity at cell-matrix adhesions. Again, we found striking differences in the organization of cell-matrix adhesions between WT and the mutant cell lines on a 12 kPa substrate (Fig. 3A–D). Larger vinculin staining was found mostly in the periphery of WT myoblasts, while it was distributed throughout the mutant cells (Fig. 3A,B). In addition, both Nespr-1^ΔKASH^ and LMNA^ΔK32^ cell lines formed larger and more numerous focal adhesions than WT myoblasts (each p < 0.005) (Fig. 3B–D). This was associated with a significant increase in vinculin (*VCL*) mRNA expression in both Nespr-1^ΔKASH^ and LMNA^ΔK32^ cell lines (Fig. 3E).

#### No modulation of spreading and contractility by Focal Adhesion Kinase (FAK) activity

Because phosphorylation of FAK is a critical regulator of rigidity-dependent strengthening of focal adhesions and cell spreading^39^, we next examined the role of FAK activity. In cells plated on 12 kPa substrates, FAK inhibition did not significantly modify the cell spreading area (Fig. 3F,G) nor the actin cytoskeleton (Suppl. Fig. 1B) in WT and mutant cells. However, FAK inhibitor inhibited cell spreading and phosphoFAK in cells plated on hard substrate (Suppl Fig. 1C,D). These data support the hypothesis that increased actin contractility in Nespr-1^ΔKASH^ and LMNA^ΔK32^ cells on a 12 kPa substrate are due to an impaired inside-outside mechanical coupling between the intra- and the extra-cellular matrix.

#### Increased traction forces in mutant cells on soft matrix

We next sought to determine whether vinculin recruitment in Nespr-1^ΔKASH^ and LMNA^ΔK32^ cells mediates focal adhesion traction on the ECM. We embedded fluorescent microbeads in 8 kPa substrates, and analysed substrate deformations due to forces exerted by cells (Fig. 3H,I). Traction forces produced by Nespr-1^ΔKASH^ and LMNA^ΔK32^ cells were significantly higher compared with those of WT (each p < 0.001), thus indicating that in Nespr-1^ΔKASH^ and LMNA^ΔK32^ cells, formation of strong adhesions was associated with higher active forces on matrix close to muscle stiffness (Fig. 3I).

#### Increased profibrotic gene expression in mutant cells

Because cell stiffness in turn modulates transcriptional programming including profibrotic genes^40^, we next investigated the effects of Nespr-1^ΔKASH^ and LMNA^ΔK32^ mutations on the expression of connective tissue growth factor (*CTGF*), collagen 1 (*COL1a*) and transforming growth factor beta (*TGFβ*). We found that Nespr-1^ΔKASH^ and LMNA^ΔK32^ myoblasts had approximately 3- to 10- fold higher mRNA expression levels of the tested profibrotic genes than WT cells (each p < 0.05, Suppl. Fig. 1E), thus suggesting that the Nespr-1^ΔKASH^ and LMNA^ΔK32^ myoblasts may affect, in the long term, the composition and the rigidity of the ECM.

### ROCK is a critical mediator of the increased actin contractility in Nespr-1^ΔKASH^ and LMNA^ΔK32^ cells

**Figure 4:**
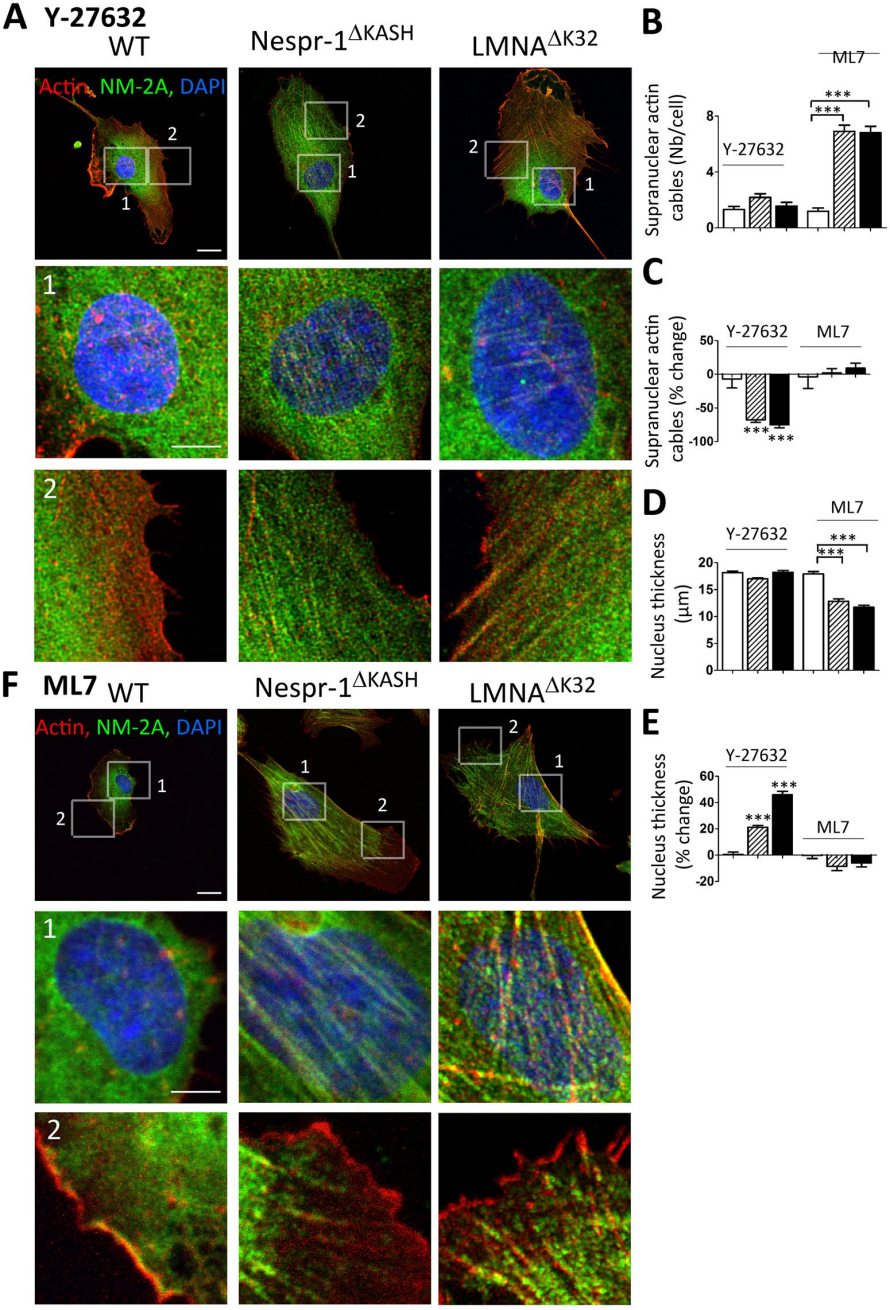
Effects of ROCK and MLCK inhibition on actin cytoskeleton in myoblasts on soft matrix (12 kPa). (**A**,**F**) Confocal images of WT, Nespr-1^ΔKASH^ and LMNA^ΔK32^ myoblasts on soft matrix and stained for F-actin (phalloidin, red) and NM-2A (green) after treatment with the ROCK inhibitor Y-27632 (**A**), and the MLCK inhibitor ML7 (**F**). Nuclei are stained with DAPI (blue). Scale bar: 10 µm. Images 1 and 2 show higher magnification of the perinuclear and periphery zones respectively. Zoom-in scale bar: 5 µm. (**B**,**C**) Supranuclear actin cable number in WT, Nespr-1^ΔKASH^ and LMNA^ΔK32^ myoblasts after treatment with Y-27632 or ML7. Values are expressed as absolute numbers (**B**) and as percent of baseline values for each cell line (**C**). (**D**,**E**) Nucleus thickness in WT, Nespr-1^ΔKASH^ and LMNA^ΔK32^ myoblasts after treatment with Y-27632 or ML7. Values are expressed as absolute numbers (**B**) and as percent of baseline values for each cell line (**C**). In (**B**–**E**), values are means ± SEM; ***p < 0.001 compared with WT (**B**,**D**) or compared to values before treatment (**C**,**E**). Only significant difference is figured.

To investigate the mechanisms involved in tensile actin stress fibre formation, we then investigated the effect of drugs acting on myosin light chain phosphorylation, namely the Y-27632 inhibitor of ROCK and the ML7 inhibitor of MLCK (Fig. 4). We found that Y-27632 reduced the number and thickness of actin stress fibres in both Nespr-1^ΔKASH^ and LMNA^ΔK32^ cell lines (Fig. 4A–C), both at the basal and apical surfaces of the mutant cells (Suppl. Fig. 1F). Further, Y-27632 significantly increased nucleus thickness in the mutant myoblasts, so that nucleus thickness did not differ in mutant and WT cells after Y-27632 (Fig. 4D,E). In contrast, the MLCK inhibitor ML7 did not significantly suppress contractile stress fibre accumulation in the mutant cells, except at the cell periphery (Fig. 4B,C,F) and did not affect nucleus thickness (Fig. 4D,E). Taken together, these results suggest that increased actomyosin contractility in mutant myoblasts plated on soft matrix is triggered by ROCK-, but not by MLCK-related pathways.

### FHOD1 mediated tensile stress fibre formation in Nespr-1^ΔKASH^ and LMNA^ΔK32^ cells

#### Increased FHOD1 expression in mutant cells

**Figure 5:**
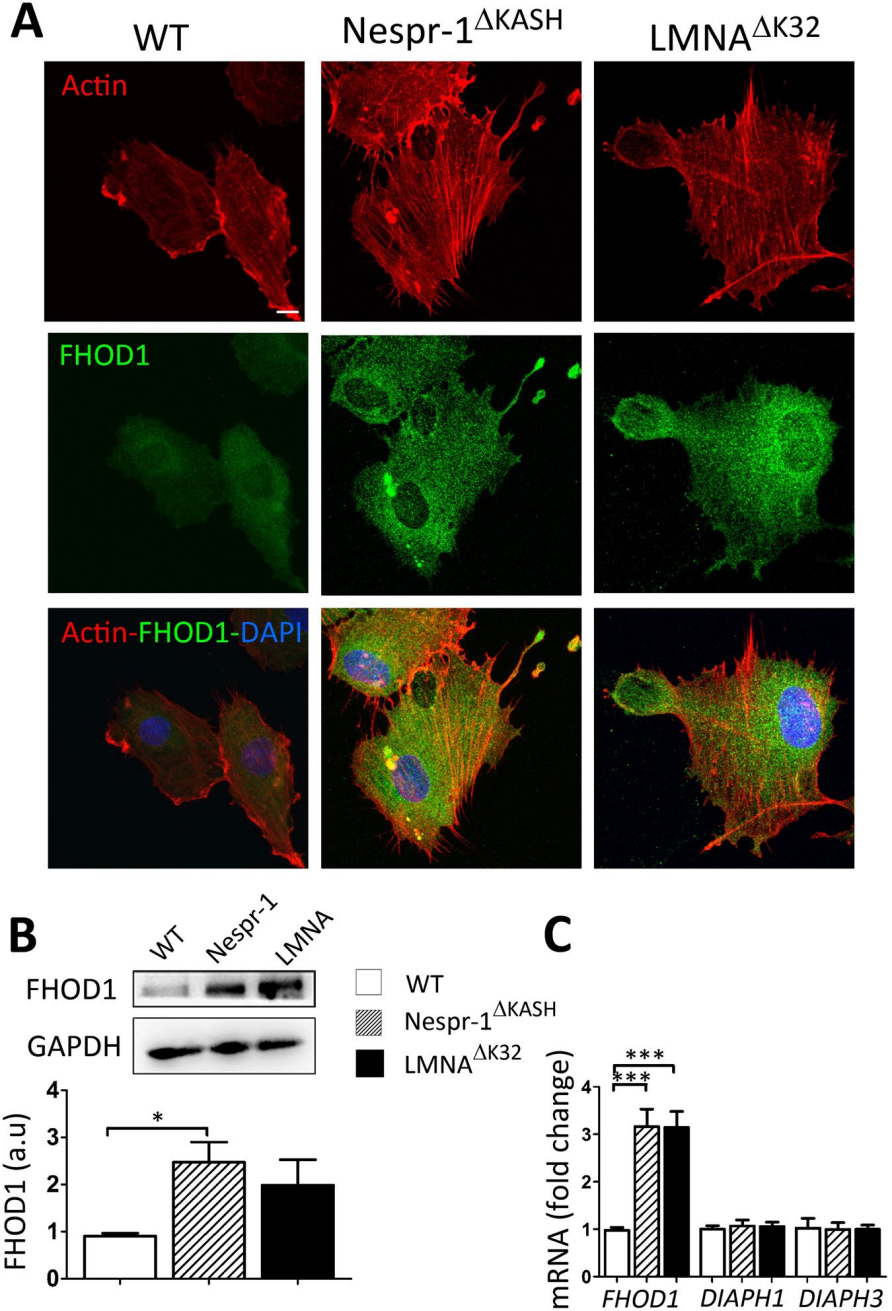
FHOD1 expression in myoblasts on soft matrix (12 kPa). (**A**) Confocal images of WT, Nespr-1^ΔKASH^ and LMNA^ΔK32^ myoblasts on soft matrix and stained for F-actin (phalloidin, red) and FHOD1 (green). Nuclei are stained with DAPI (blue). (**B**) Representative western-blot of FHOD1 in WT, Nespr-1^ΔKASH^ and LMNA^ΔK32^ myoblasts cultured on soft substrates. Histogram represents FHOD1 quantifications obtained in WT, Nespr-1^ΔKASH^ and LMNA^ΔK32^ myoblasts and normalized by GAPDH. Values are means ± SEM, n = 6 in WT, Nespr-1^ΔKASH^ and LMNA^ΔK32^ myoblasts; *p < 0.05. Only significant difference is figured; au: arbitrary units. (**C**) Histogram represent mRNA concentrations of *FHOD1*, *DIAPH1* and *DIAPH3* normalized to *β2 microglobulin* expression and expressed in arbitrary units (au). Values are means ± SEM, n = 5 in WT, Nespr-1^ΔKASH^ and LMNA^ΔK32^ myoblasts, ***p < 0.001 compared with WT. Only significant difference is figured.

We next investigated whether formins, which are potent regulators of actin assembly and cytoskeletal remodelling^41^, may contribute to the accumulation of contractile stress fibres in mutant cells plated on a soft substrate. Because the formin FHOD1 induces stress fibre formation in a ROCK dependent manner^42, 43^, we hypothesized that aberrant rigidity response in the mutant myoblasts could be related to abnormal FHOD1 activity. FHOD1 staining was more pronounced in Nespr-1^ΔKASH^ and LMNA^ΔK32^ myoblasts plated on soft matrix than in WT (Fig. 5A). Furthermore, both the mRNA expression and the protein levels of FHOD1 were higher in mutant myoblasts compared with WT, although FHOD1 protein expression only reached significance for Nespr-1^ΔKASH^ (Fig. 5B,C). In contrast, the mRNA levels of the two major diaphanous-related formins *DIAPH1* and *DIAPH3* did not significantly differ in the Nespr-1^ΔKASH^ and LMNA^ΔK32^ myoblasts compared with WT (Fig. 5C).

#### Rescue of mutant cell morphology by inhibition of FHOD1 activity and knock-down

**Figure 6:**
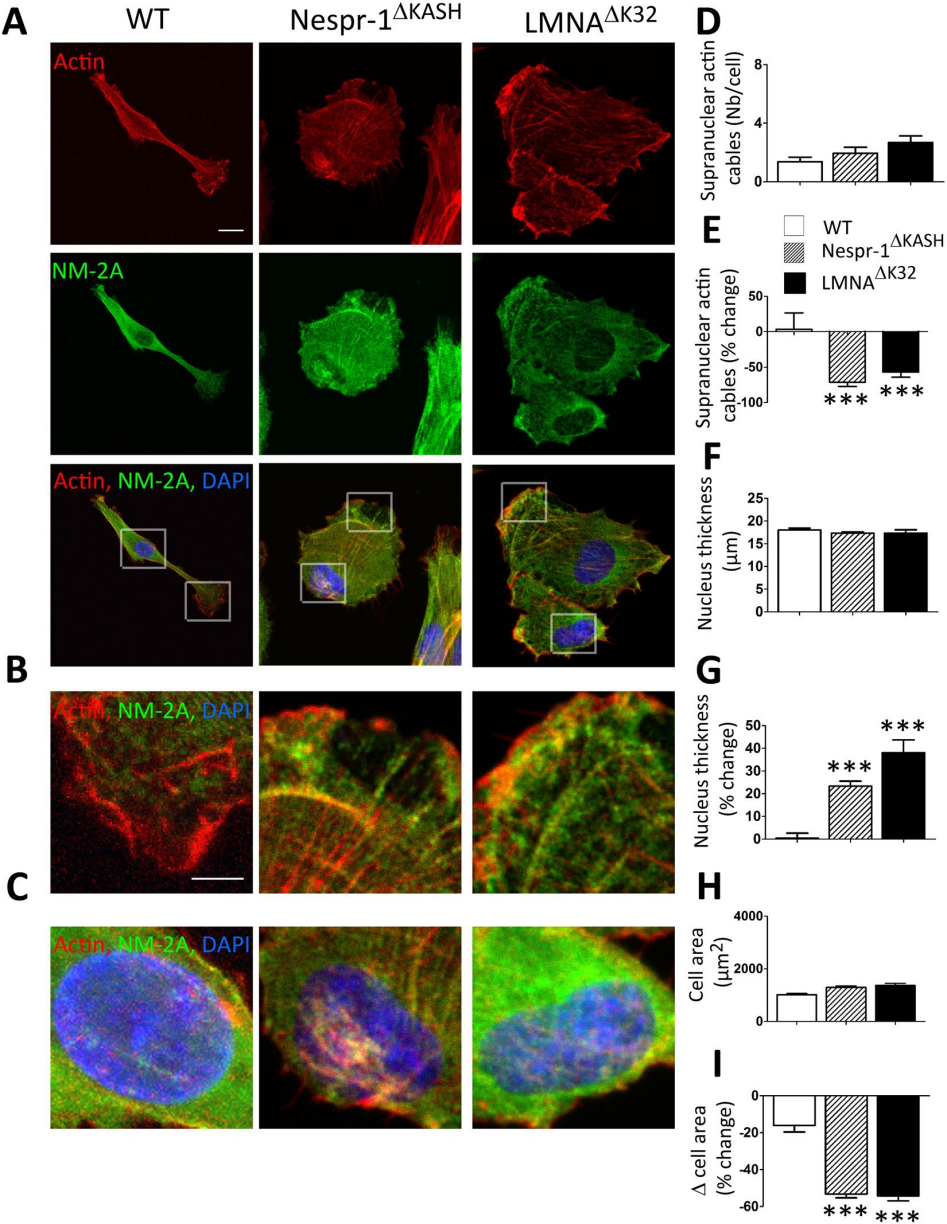
siRNA against FHOD1 reduced actin contractility and nucleus thickness in myoblasts on soft matrix (12 kPa). (**A**) Confocal images of WT, Nespr-1^ΔKASH^ and LMNA^ΔK32^ myoblasts on soft matrix and stained for F-actin (phalloidin, red) and NM-2A (green) after siRNA against FHOD1. Scale bar: 10 µm (**B**,**C**) Zoom-in of actin cytoskeleton at cell periphery (**B**) and in the perinuclear regions (**C**). Scale bar: 10 µm. (**D**,**E**) Supranuclear actin cable number in WT, Nespr-1^ΔKASH^ and LMNA^ΔK32^ myoblasts after siRNA against FHOD1. Values are expressed as absolute numbers (**D**) and as percent of baseline values for each cell line (**E**). Values are means ± SEM, n ≥ 19 in WT, Nespr-1^ΔKASH^ and LMNA^ΔK32^ myoblasts, ***p < 0.001 compared to values before siRNA against FHOD1. Only significant difference is figured. (**F**,**G**) Nuclear thickness in WT, Nespr-1^ΔKASH^ and LMNA^ΔK32^ myoblasts after siRNA against FHOD1. Values are means ± SEM. n ≥ 19 in WT, Nespr-1^ΔKASH^ and LMNA^ΔK32^ myoblasts, ***p < 0.001 compared to values before siRNA against FHOD1. Only significant difference is figured. (**H**,**I**) Cell spreading area in WT, Nespr-1^ΔKASH^ and LMNA^ΔK32^ myoblasts after siRNA against FHOD1. Values are expressed as absolute values (**H**) and as percent of baseline values for each cell line (**I**). Values are means ± SEM, n ≥ 50 cells in each line; ***p < 0.001 compared to values before siRNA against FHOD1. Only significant difference is figured.

Treatment with the “small-molecule inhibitor of formin homology 2 domain” (SMIFH2)^44^ inhibited spreading and suppressed actin stress fibres in WT and mutant myoblasts (Suppl Fig. 1G). To further investigate the role of FHOD1 on the accumulation of contractile stress fibres, we knocked-down FHOD1 in WT and mutant myoblasts using small interfering RNA (siRNA) (Suppl. Fig. 1H, Fig. 6). In Nespr-1^ΔKASH^ and LMNA^ΔK32^ cell lines, siRNA-mediated depletion of FHOD1 largely rescued cell morphology, as judged by the large reduction of actomyosin stress fibre accumulation in the perinuclear region, and reappearance of transverse arcs at the cell periphery (Fig. 6A,D,E). In addition, FHOD1 loss restored the nuclear thickness (Fig. 6F,G) and significantly reduced the cell spreading area in the mutant but not in WT cells (Fig. 6H,I). Taken together, these data indicate that FHOD1 is a key actor of actin stress fibre accumulation in Nespr-1^ΔKASH^ and LMNA^ΔK32^ myoblasts plated on matrix stiffness close to that of muscle.

### Discrete defects of Nespr-1^ΔKASH^ and LMNA^ΔK32^ cells plated on hard substrate

**Figure 7:**
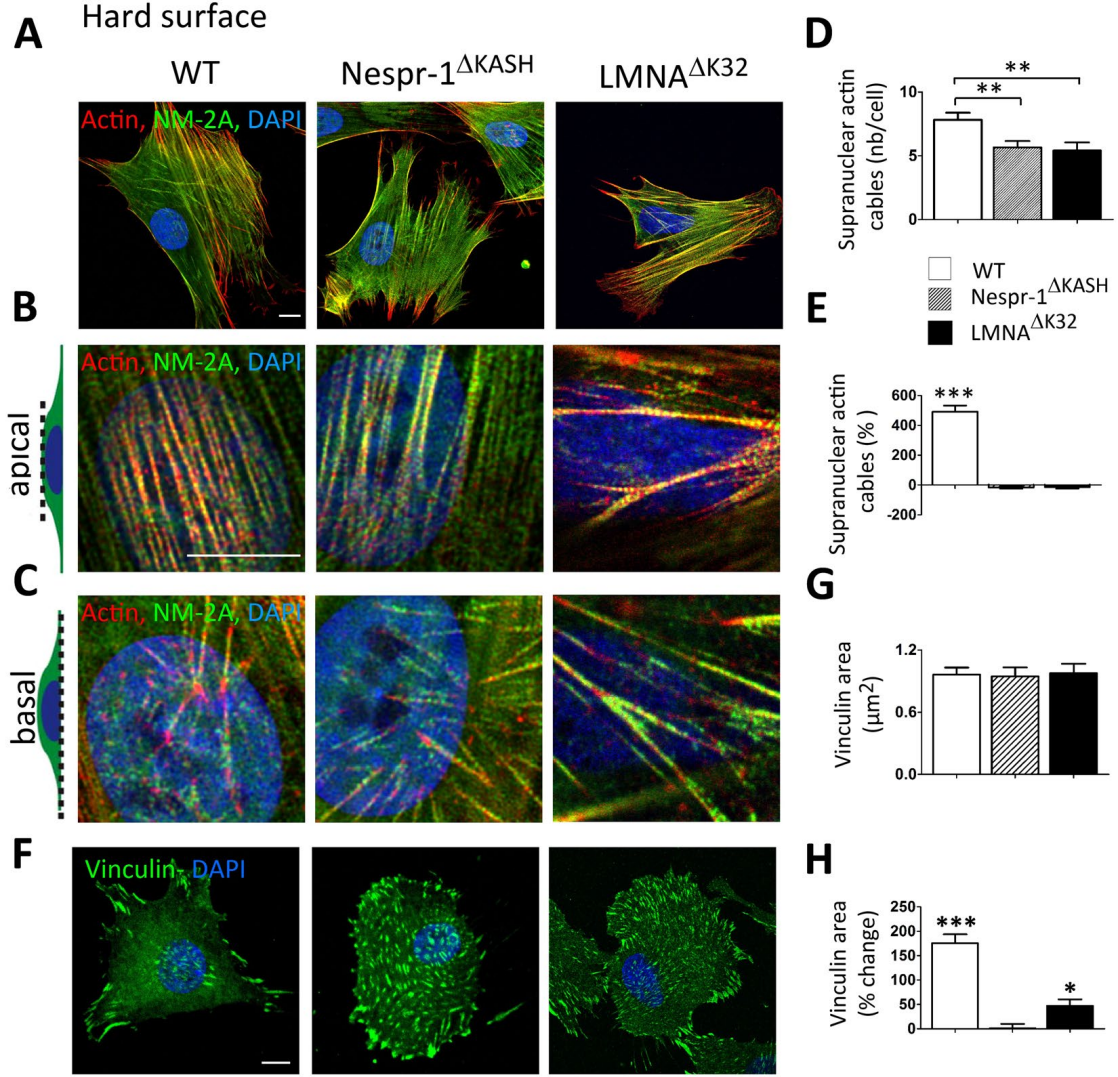
Impaired ability to sustain high external force in Nespr-1^ΔKASH^ and LMNA^Δ**K32**^ cells. (**A**) Confocal images of WT, Nespr-1^ΔKASH^ and LMNA^ΔK32^ myoblasts on fibronectin-coated rigid matrix (glass) and stained for F-actin (phalloidin, red) and non-muscle myosin IIA (NM-2A, green). Nuclei are stained with DAPI (blue). Scale bar: 10 µm. (**B**,**C**) Zoom-in of actin cytoskeleton in the perinuclear regions. Confocal images were taken at the apical (**B**) and basal (**C**) surface of the cell. (**D**,**E**) Supranuclear actin cable number on hard surface in WT, Nespr-1^ΔKASH^ and LMNA^ΔK32^ myoblasts. Values expressed as absolute values (**D**) and as percent changes versus values obtained at 12 kPa (**E**). Values are means ± SEM. n ≥ 20 in WT, Nespr-1^ΔKASH^ and LMNA^ΔK32^ myoblasts, ***p < 0.01 vs WT. Only significant difference is figured (**F**–**H**) Cell matrix adhesions on hard surface. (**F**) Confocal images of WT, Nespr-1^ΔKASH^ and LMNA^ΔK32^ myoblasts on hard matrix (glass) and stained with antibody against vinculin (green). Nuclei are stained with DAPI (blue). Scale bar: 10 µm. (**G**,**H**) Histograms of vinculin area in WT, Nespr-1^ΔKASH^ and LMNA^ΔK32^ myoblasts, expressed as absolute values (**G**) and as percent changes versus values obtained at 12 kPa (**H**). At least 50 cells of each type were analysed. *p < 0.05 and ***p < 0.001 compared with corresponding value obtained on 12 kPa substrate. Only significant difference is figured.

The capacity of Nespr-1^ΔKASH^ and LMNA^ΔK32^ myoblasts to adapt to rigid, non-compliant substrates was then determined by examination of the organization of contractile actin cytoskeleton in cells plated on glass (Fig. 7). WT myoblasts cultured on glass exhibited larger and more abundant actin stress fibres at the apical surface of the nucleus, compared to soft substrate (Fig. 7A–E). In contrast, matrix rigidity had no significant impact on the number of contractile actin cable bundles in Nespr-1^ΔKASH^ and LMNA^ΔK32^ (Fig. 7E). In addition, supranuclear actin bundles appeared disorganized in lamin and nesprin mutant cells (Fig. 7A,B), as previously reported in mouse cells with disrupted nuclear-cytoskeletal linkages^25^. In WT plated on glass, the area occupied by focal adhesions was 1.8 fold higher compared with soft matrix (Fig. 7G,H). In contrast, the area occupied by focal adhesions did not differ in Nespr-1^ΔKASH^ and there was only a 0.5 fold increase in the area occupied by focal adhesions in LMNA^ΔK32^ (Fig. 7F,H) in hard compared to soft ECMs. Thus, on stiff substrates, the only visible effect is a slight reduction of the perinuclear actin cytoskeleton in Nespr-1^ΔKASH^ and LMNA^ΔK32^ cells, as previously reported^25, 45^.

## Discussion

In this study, we analysed whether pathogenic lamin and nesprin mutations responsible for severe muscle disorders impaired the mechanical coupling between the cell interior and the ECM. On soft matrix with a stiffness close to that of muscle^37^, *LMNA* or *SYNE-1* mutations increased intracellular contractility, resulting in abnormal force coupling between the actin cytoskeleton and the ECM. Furthermore, we identified FHOD1 as an important regulator of actin cytoskeleton organization in nesprin-1 and lamin mutant muscle cell precursors. To date, all mutations in A-type lamins or nesprins that cause muscular dystrophies have been shown to compromise the nesprin/SUN/lamin interactions, resulting in impaired nucleo-cytoskeletal linkages^9, 16, 18, 20^. Therefore, we propose that the integrity of A-type lamins and nesprin-1 is required to modulate the activity of the formin FHOD1 and to regulate inside to outside signalling by which muscle cell precursors adapt their intracellular tension to the softness of their native extracellular microenvironment, presumably through an impaired functional integrity of nuclear-cytoskeleton linkages.

In cells plated on glass or plastic, mechanical forces from the ECM induce conformational changes in sarcolemmal/transmembrane receptor proteins and focal adhesions that activate outside-inside pathways and generate cytoskeletal tension^46^. When plated on a rigid substrate, Nespr-1^ΔKASH^ and LMNA^ΔK32^myoblasts had a looser and more irregular perinuclear actin cytoskeleton (Fig. 7B), consistent with previous data on cells with disrupted LINC complex^20, 21, 24, 47, 48^ or deficient for lamin A/C^24, 25, 49^. Taken together, these data support the hypothesis that Nespr-1^ΔKASH^ and LMNA^ΔK32^ myoblasts are unable to sustain high external mechanical challenges.

Softer substrate makes it possible to analyse cell tension behaviour in a more physiological context and to test inside to outside mechanosensing pathways. We found that Nespr-1^ΔKASH^ and LMNA^ΔK32^ myoblasts plated on soft substrates exhibited accumulation of contractile actin stress fibres (Fig. 2A–D), increased traction force (Fig. 3H,I), and thinner nuclei compared with controls (Fig. 2E). Consistently, we previously reported that lamin mutant myoblasts are defective to respond to 3D softer surroundings^29^. Here, we have shown that inhibiting FAK phosphorylation did not modify the spreading area or the actin cytoskeleton in LMNA and nesprin-1 mutant myoblasts plated on a soft substrate (Fig. 3G and Suppl Fig. 1B). Thus, increased internal tension in mutant cells appears to be independent of outside-in signals from the ECM. Thus, it is plausible that nesprin-1 and A-type lamin mutations impaired a feedback mechanism between the tension of the inside of the cell and the elasticity of the extracellular environment, presumably through dysfunction of nuclear-cytoskeletal linkages.

We previously implicated abnormal Yes-Associated Protein (YAP) activity in the abnormal mechanosensing response of lamin mutant myoblasts^29^. Precise molecular mechanisms by which ECM stiffness and cell shape control YAP activity still remain unknown. However, an obvious candidate for YAP regulation is the actin cytoskeleton itself, given that increased F-actin polymerization has been shown to increase YAP activity^50^.

A rich variety of regulators assembles, maintains and disassembles actin cytoskeletal structures. Among these, DRFs constitute a family of Rho-GTPase-regulated proteins that modulate actin and microtubule cytoskeletons^30^ in a force-sensitive fashion^31, 32^. Contrary to most DRFs that stimulate nucleation and/or elongation of actin filaments, phosphorylation of the formin FHOD1 by the Rho effector kinase ROCK^42, 43^ disrupts the auto-inhibitory state of FHOD1 and promotes formation of linear F-actin stress fibres^42, 51^, as observed in our study. In contrast, ARP2/3 activity typically produces branched F-actin structures. Interestingly, inhibition of formins down-regulates YAP activity whereas small-molecule inhibitors of ARP2/3 activity are ineffective^52^. Therefore, one can assume that FHOD1-dependent F-actin accumulation contributes to YAP activation and mechanosensing defects in lamin and nesprin-1 mutant cells plated on a soft matrix.

Inhibiting FHOD1 activity critically supported its contribution in the abnormal coupling between the cell interior and the soft matrix. We showed that FHOD1 depletion by siRNA (Fig. 6) and/or inactivation with the ROCK inhibitor Y-27632 (Fig. 4A–C) or with the pan-formin inhibitor SMIFH2 (Suppl Fig. 1G) strongly inhibited the accumulation of contractile actin fibres in mutant cells plated on soft matrix.

Interestingly, FHOD1 is known to interact with the giant nesprin-2, and may contribute to stabilize TAN lines by crosslinking adjacent nesprin proteins^33^. In addition, it has been proposed that connecting nesprin-2 to actin cables through FHOD1 modulates FHOD1 activity, as interactions between the FHOD1 N-terminus with its C-term auto-inhibitory domain or with activated GTPases can affect FHOD1-nesprin-2 interaction^33^. Given the known interaction of nesprin-2 with both nesprin-1 and A-type lamins, we propose that in human muscle cell precursors, nesprin-1 and lamin mutations may directly or indirectly alter the interactions of FHOD1 with nesprin-2, leading to an increased activity of FHOD1 in soft matrix. This in turn promotes the formation of an FHOD1-dependent pool of F-actin that contributes to abnormal YAP activity and defective mechanosensing response.

In conclusion, we describe a previously unrecognized role for lamins and nesprin-1 in the pathways regulating sensing of substrate stiffness in human muscle cell precursors. Our results implicate lamins and nesprin-1 in regulating a feedback loop that couples the nucleus to the rigidity of the extracellular microenvironment, through inside to outside pathways involving the actin cytoskeleton and the formin FHOD1. Defective regulation of mechanosensing in myopathic cells uncouples the actin cytoskeleton from the force generated by the extracellular muscle environment and therefore might contribute to the progression of muscle disease.

## Materials and Methods

### Human myoblasts and cell culture

Muscle biopsies were obtained from the Bank of Tissues for Research (BTR, a partner in the EU network EuroBioBank) in accordance with European recommendations and French legislation. Written informed consent was obtained from all patients. Experimental protocols were approved by our institution (INSERM). Experiments were performed using immortalized human myoblasts carrying the following *LMNA* or *SYNE-1* mutations responsible for severe congenital muscle disorders^34, 36^: heterozygous *LMNA*c.94_96delAAG, p.Lys32del (hereafter referred to as *LMNA* ^ΔK32^), *SYNE-1* homozygous c.23560 G<T, p.E7854X leading to a stop codon in exon 133 and deletion of the carboxy-terminal KASH domain (hereafter referred to as Nespr-1^ΔKASH^). Control immortalized myoblasts were obtained from one healthy control subject without muscular disorders (hereafter referred to WT). All experiments were performed in accordance with the French legislation on ethical rules. Immortalized cell lines were obtained by transducing cells with pBABE retroviral vectors carrying Cdk4 and hTERT, as previously described^53^. Primary and immortalized myoblasts were cultured in growth medium, as previously described^29^. Primary and stable cell lines from the same patient invariably showed the same phenotypes, but immortalized cell lines overcome the problem of limited proliferation and cellular senescence present in primary myoblasts. The localization of nuclear envelope proteins in WT, LMNA^ΔK32^ and Nespr-1^ΔKASH^ myoblasts is depicted in Suppl Fig. 2. In mutant myoblasts, emerin, nesprin-2, lamin A/C, and SUN2 were localized at the nuclear rim, as observed for the WT cells, although nucleoplasmic localization of lamin A/C and SUN-2 was also present in LMNA^ΔK32^ and Nespr-1^ΔKASH^, respectively. Nesprin-1 was localized to the nuclear envelope in both WT and LMNA^ΔK32^ myoblasts. In Nespr-1^ΔKASH^ myoblasts, immunostaining with either MANNES1A or N1G-7C8 which recognizes the C-term and epitopes preceding the start of the β1 sequence, respectively^54^, show only very weak background staining, consistent with the fact that truncated mutant protein has not been detected in the Nespr-1^ΔKASH^ myoblasts^35^.

Cell cultures were performed on 2D conventional rigid substrates, and on soft hydrogels. Easy coated polyacrylamide (PAM) hydrogels of 12 kPa Young’s modulus (E) in 35 mm Petrisoft (plastic bottom) and Softview (glass bottom) cell culture dish formats were purchassed (Matrigen, Brea, California, USA). Polydimethylsiloxane (PDMS) substrates of 5 kPa, 20 kPa and 700 kPa were prepared from the commercially available Sylgard 184 silicone elastomer kit (Down Corning) by mixing base and the curing agent in varying ratios, as described previously^55^. Specifically, PDMS with base to crosslinker (w/w) ratio of 75:1, 50:1 and 25:1 were prepared to obtain 5 kPa, 20 kPa and 700 kPa^55^. All surfaces were coated with fibronectin at a concentration of 10 µg/ml (Sigma-Aldrich, Saint Quentin-Fallavier, France). Before cell plating, plates were washed with PBS and growth medium.

### Drug treatment and siRNA

ROCK inhibitor Y-27632, myosin light chain kinase inhibitor ML7, the formin inhibitor SMIFH2 and the inhibitor of focal adhesion kinase (FAK) phosphorylation 14 (also known as 1,2,4,5-Benzenetetraamine 4HCl or Y15) were diluted to final concentrations of 10, 20, 25 and 2.5 µM respectively in the culture medium. Cells were incubated with each drug for 6 hours except for the FAK inhibitor where cells were incubated with the drug for 2 hours. siRNA transfections were done with HiPerfect (Qiagen, Venlo, Netherlands) according to manufacturer’s instructions. Downregulation of FHOD1 was observed 72 h after transfection. Sequences of siRNAs are provided in Supplementary Table 1. FAK inhibition reduced the staining of pFAK and significantly reduced the spreading area (each p < 0.001) in the 3 cell lines plated on hard surface (Suppl Fig. 1B and C), thus attesting to the efficacy of FAK inhibition.

### Immunocytochemistry

Myoblasts were fixed for 5 min with 4% formaldehyde, permeabilized with 0.5%Triton X100 and blocked with 5% bovine serum albumin (BSA) diluted in PBS. Myoblasts were stained with Phalloidin-Alexa 568 to label F-actin (Interchim, Montluçon, France). The following primary antibodies were used for immunostaining: anti-vinculin (Sigma-Aldrich, Saint Quentin-Fallavier, France), anti-non muscle myosin IIA (NM-2A) (Abcam, Paris, France), anti-lamin A/C (sc-6215, Santa Cruz Biotechnology, Santa Cruz, California, USA), anti-emerin (NCL-emerin Novocastra), and anti-SUN2^11^ (generously provided by D. Hodzic), anti-nesprin 1 (N1G-7C8 and MANNES1A), which recognize exons 84-85 and exons 143-146 of nesprin-1, respectively)^54^, anti-nesprin-2 (MANNES2A)^54^, and anti FHOD1 (ab73443, Abcam). Secondary antibodies (Life technologies, Saint-Aubin, France; 1/500) were: Alexa Fluor 488 goat anti-mouse IgG, Alexa Fluor 568 goat anti-rabbit IgG, Alexa Fluor 488 donkey anti-mouse IgG, or Alexa Fluor 488 rabbit anti-goat IgG. The preparations were mounted on slides with fluorescent mounting medium containing DAPI (Vectashield, Vector Labs, Berlingame, California). Confocal images were taken with an Olympus FV 1000 (Olympus, Hamilton, Bermuda) and a Leica SP2 (Leica Microsystems, Wetzlar, Germany) microscopes.

### Image analysis

All image analyses were performed using ImageJ software (version 1.51). Localization of nuclear envelope proteins was determined from one image centred on the middle of the nucleus. Stress fibre number was determined by drawing a 4 pixel-width line oriented perpendicular to the long axis of the cell, 5 µm apart from the nucleus, and determining the grey intensity profiles, where the x-axis represents the distance through the selection and the y-axis the averaged pixel intensity. The number of actin fibres was counted as number of positive peaks in this profile. Vinculin area and the number of focal adhesions per cell were measured from Z-stack images. For measuring the nuclear height and volume, z-stacks were taken at an interval of 0.3 µm and the x-z view projections were reconstructed using ImageJ. The height was calculated as the distance between the top and bottom nuclear edge, from at least 12 cells per condition. Cell spreading was measured by quantifying the area of at least 50 cells/line from four random fields for each cell conditions.

### SDS-PAGE and protein analysis

Proteins were extracted in total protein extraction buffer (50 mM Tris-HCl, pH 7.5, 2% SDS, 250 mM sucrose, 75 mM urea, 1 mM DTT) including protease inhibitor (25 μg/ml Aprotinin, 10 μg/ml Leupeptin, 1 mM 4-(2-aminoethyl)-benzene sulfonyl fluoride hydrochloride and 2 mM Na_3_VO_4_). Total protein extracts (20 µg) were separated on 10% SDS-PAGE and transferred on 0.45 µm nitrocellulose membranes. Membranes were blocked in 5% skim milk in TBS-tween20 and hybridized with anti-FHOD1 (ab 73443, Abcam), and anti-GAPDH (Santa Cruz biotechnology, Santa Cruz, California, USA) antibodies and with either secondary HRP-conjugated sheep anti-mouse, or donkey anti-goat IgG (Jackson ImmunoResearch). Immunoblots were visualized with Immobilon Western Chemiluminescent HRP Substrate (Millipore, Molsheim, France) on a G-Box system with GeneSnap software (Ozyme, Saint Quentin, France). Quantification was performed using ImageJ. GAPDH was used as loading control.

### Quantification of gene expression

RNeasy mini kit (Qiagen, CourtabNoeuf, France) was used to prepare total RNA. For reverse transcription and quantitative RT-PCR, Superscript III (Life technologies, Saint-Aubin, France) with random primers was used for cDNA generation and SYBR Green PCR Master Mix was used according to manufacturer instructions. Experiments were performed on Light Cycler 480 System (Roche, Meylan, France) with each sample performed in triplicate. *RPLPO* or β2-microglobulin were used as housekeeping genes. Primer sequences are listed in Supplementary Table 2.

### Traction force microscopy

Fibronectin coated polyacrylamide gels (Young’s modulus of 8 kPa) for traction force microscopy were prepared on glass bottom dishes as described previously^56, 57^. Red fluorescent microspheres (0.2 μm diameter) were suspended in the polyacrylamide gel before gel formation and used as fiduciary markers. Myoblasts were plated at low concentrations (5% confluence) and incubated for 12 h at 37 °C with 5% CO_2_ in full growth media. Experiments require images from cells (DIC images) and red fluorescent images of beads at the same time. Imaging was performed every 5 min, and continued for 90 min. After the acquisition of images, trypsin (Gibco) was added to the culture medium to detach cells from the gel. Reference images of fluorescent beads at the “relaxed” state were taken ∼10 min thereafter. Displacements are obtained by calculating the difference between the present and the reference position. Stresses exerted by cell are calculated by solving the inverse problem of traction force microscopy (TFM) using the adjoint method formulated in the finite elements framework by minimizing the Tikhonov functional^58^. Then stresses are computed using the protocol described previously^59^ using finite element method coupled with the regularized inverse problem, i.e. the so-called adjoint method^60^.

### Statistical analysis

Graphpad Prism (Graphpad Software, La Jolla, California) was used to calculate and plot mean and standard error of the mean (SEM) of measured quantities. Statistical significances were assessed by ANOVA followed by Bonferroni or two-tailed unpaired t-tests. Differences between conditions were considered significant at p < 0.05.

## Electronic supplementary material

Supplementary data
