# Supplementary material for: Lamins and nesprin-1 mediate inside-out mechanical coupling in muscle cell precursors through FHOD1
Source: Sci Rep. 2017 Apr 28;7:1253. doi: 10.1038/s41598-017-01324-z (PMC5430732; doi:10.1038/s41598-017-01324-z)
Supplement: Supplementary file 1 — Supplementary data [file 41598_2017_1324_MOESM1_ESM.pdf]

Lamins and nesprin-1 mediate inside-out mechanical coupling in muscle cell precursors through FHOD1

[1] Christine Schwartz $, [1] Martina Fischer $, [1] Kamel Mamchaoui, [1] Anne Bigot, [2,3] Thevy Lok, [2,3] Claude Verdier, [4,5] Alain Duperray, [2,3] Richard Michel, [6] Ian Holt, [1] Thomas Voit ($$),[7] Suzanna Quijano-Roy, [1] Gisèle Bonne, [1] Catherine Coirault *.

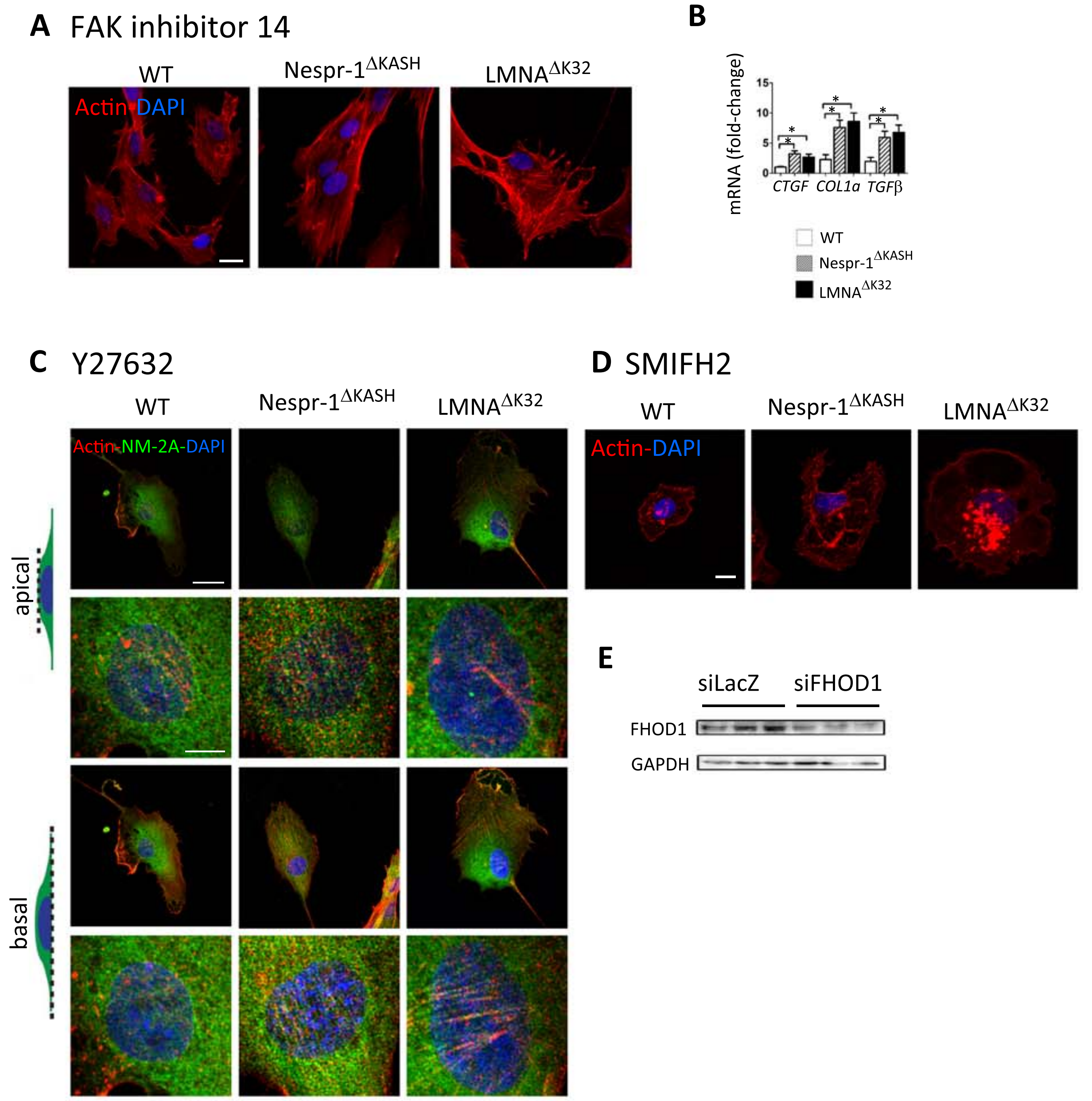

Suppl Fig. 1. (A) Effect of the inhibitor of the FAK phosphorylation 14 on actin cytoskeleton in myoblasts on 12 kPa matrix. Confocal images of WT, Nespr-1$^{\Delta KASH}$ and LMNA$^{\Delta K32}$ myoblasts on soft matrix and stained for F-actin (phalloidin, red) and DAPI (blue). Scale bar: 20 µm **(B)** Histogram of *CTGF*, *COL1a* and *TGFβ* mRNA expression in WT, Nespr-1$^{\Delta KASH}$ and LMNA$^{\Delta K32}$ myoblasts. Values are means ± SEM; *p<0.05 compared with WT and expressed in arbitrary units (au). Values are means ± SEM, n=5 in each cell lines from 2 separate experiments. **(C)** Effect of the ROCK inhibitor Y-27632 on apical and bottom actin cytoskeleton in myoblasts on soft matrix. Confocal images of WT, Nespr-1$^{\Delta KASH}$ and LMNA$^{\Delta K32}$ myoblasts on soft matrix and stained for F-actin (phalloidin, red) and NM-2A (green) after treatment with Y-27632. Nuclei are stained with DAPI (blue). Apical (left) and bottom (right) views. Scale bar: 10 µm. **(D).** Effect of the pan formin inhibitor SMIFH2 on actin cytoskeleton in myoblasts on soft matrix. Confocal images of WT, Nespr-1$^{\Delta KASH}$ and LMNA$^{\Delta K32}$ myoblasts on soft matrix and stained for F-actin (phalloidin, red). Scale bar: 10 µm. **(E)** Representative western-blot of FHOD1 after treatment with siRNA against LacZ or against FHOD1.

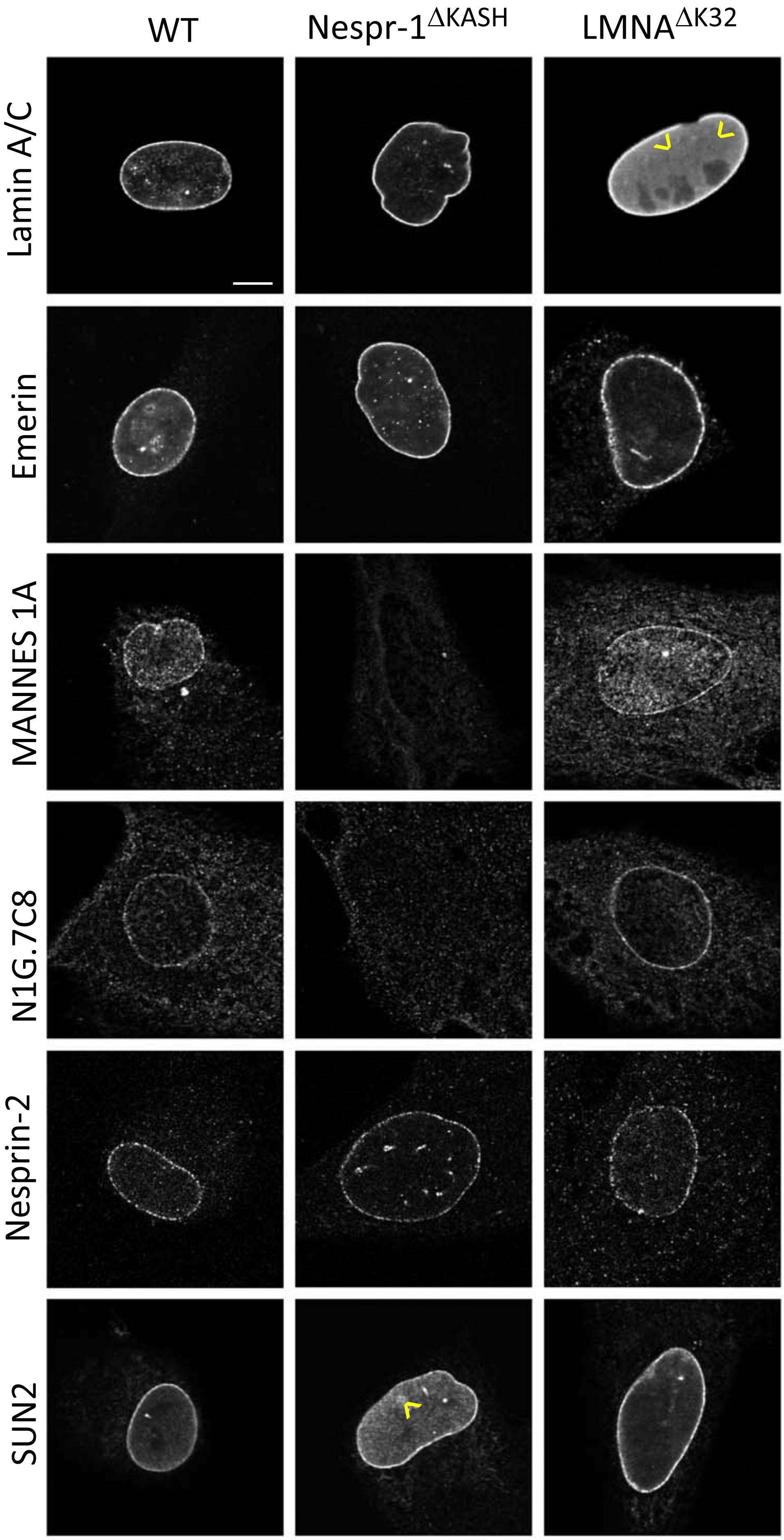

Suppl Fig. 2. Nuclear morphology of WT, Nespr-1$^{\Delta KASH}$ and LMNA$^{\Delta K32}$ myoblasts. Confocal immunofluorescence images were obtained from 1 image centered on the middle of the nucleus. Emerin and nesprin-2 (MANNES2A) staining revealed no defect in localization to the nuclear envelope. Arrowheads highlight examples where lamin A/C or SUN2 redistribute to the nucleoplasm. MANNES1A: nesprin-1 antibody against the C-term part of the nesprin-1; N1G-7C8: nesprin-1 antibody targeted against exons 84-85. Nesprin-1 antibodies show only very weak background staining in Nespr-1$^{\Delta KASH}$ myoblasts. Scale bar: 5 µm.

Supplementary Table 1. List of siRNA

siRNA-FHOD1	GCCACUGUUUGACCUGAAA
siRNA-LacZ	CCACCAAGCUAGAUAAAGA

Supplementary Table 2. List of target genes and primers

Target genes	Primer forward	Primer reverse
CTGF	ACCGACTGGAAGACACGTTTG	CCAGGTCAGCTTCGCAAGG
DIAPH1	GTTGCAGGACCTTCGAGAGA	CCGGCACTTGAAGTCAGGAT
DIAPH3	GCGGTATGCATTGTAGGGGA	CAGGAGATGTAACCAGGGCA
FHOD1	GCATTGAGAAGCTACTGACC	CATTCTGTACCAGCTGTTCC
MYH9	GGCAGGGCACGGAAGGCTAAG	AACAGGCGCTGCTTCTCCCGA
COL1a	AAGAGGAAGGCCAAGTCGAG	CACACGTCTCGGTCATGGTA
TGFβ	CGCGTGCTAATGGTGGAAAC	GTTCAGGTACCGCTTCTCGG
VCL	TCGCAAATGGTCCAGCAAGGGC	CGCTTGGTACCACTGCCCCC
B2M	AGATGAGTATGCCTGCCGTG	GCGGCATCTTCAAACCTCCA
RPLP0	CTCCAAGCAGATGCAGCAGA	ATAGCCTTGCGCATCATGGT
